# Role of Oct4 in the early embryo development

**DOI:** 10.1186/2045-9769-3-7

**Published:** 2014-04-29

**Authors:** Guangming Wu, Hans R Schöler

**Affiliations:** 1 Department of Cell and Developmental Biology, Max Planck Institute for Molecular Biomedicine, Röntgenstrasse 20, 48149 Münster, Germany; 2 Medical Faculty, University of Münster, Domagkstr. 3, 48149 Münster, Germany

**Keywords:** Oct4, Oct4B, Totipotency, Pluripotency, Embryo, Development

## Abstract

Oct4 is a key component of the pluripotency regulatory network, and its reciprocal interaction with Cdx2 has been shown to be a determinant of either the self-renewal of embryonic stem cells (ESCs) or their differentiation into trophoblast. Oct4 of maternal origin is postulated to play critical role in defining totipotency and inducing pluripotency during embryonic development. However, the genetic elimination of maternal *Oct4* using a Cre-lox approach in mouse revealed that the establishment of totipotency in maternal Oct4–depleted embryos was not affected, and that these embryos could complete full-term development without any obvious defect. These results indicate that Oct4 is not essential for the initiation of pluripotency, in contrast to its critical role in maintaining pluripotency. This conclusion is further supported by the formation of *Oct4*-GFP– and Nanog- expressing inner cell masses (ICMs) in embryos with complete inactivation of both maternal and zygotic *Oct4* expression and the reprogramming of fibroblasts into fully pluripotent cells by *Oct4*-deficient oocytes.

## Introduction

**Figure 1:**
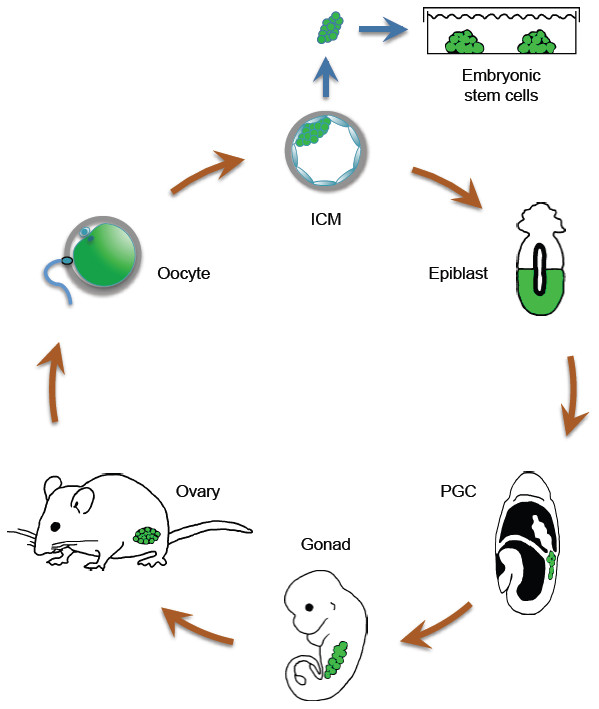
**Oct4 expression during the mouse life cycle.** Cells and tissues expressing Oct4 are marked in green. Oct4 is expressed in mouse oocytes as a maternal transcript and protein. Zygotic Oct4 expression is activated prior to the 8-cell stage and is abundant and uniform in all cells of the embryo throughout the morula stage. However, as the outer cells of the embryo differentiate into the TE, Oct4 expression is restricted to cells of the ICM in the blastocyst. After implantation, Oct4 expression is maintained in the epiblast. Finally, Oct4 expression becomes restricted to primordial germ cells (PGCs), which are first specified in the extraembryonic mesoderm at the base of the allantoic bud during gastrulation. PGCs give rise to gametes, which following fertilization will develop into a new organism of next generation. Oct4 expression is based on data previously reported by several studies [6, 7, 9–11].

Life is like a journey of torch relay. From generation to generation, our bodies vanish at the end of our lives, but the germ cells are passed on to the next generation, ensuring the continuity and prosperity of our species. In comparison with the somatic cells, these germ cells possess many unique properties, of which the expression of Oct4 is the most important as it is required for the survival of primordial germ cells (PGCs) [1, 2]. *Oct4* is also expressed specifically in the inner cell mass (ICM) and embryonic stem cells (ESCs), the cells derived from the ICM [3]. Interestingly, *Oct4* is expressed in mouse oocytes as a maternal transcript and protein [1, 4–6]. As is typical for most maternal mRNAs, levels of *Oct4* mRNA drop dramatically after fertilization [6]. Zygotic *Oct4* expression is activated prior to the 8-cell stage, with a significant increase in both mRNA and protein levels [4, 6]. *Oct4* expression is abundant and uniform in all cells of the embryo throughout the morula stage. However, as the outer cells of the embryo differentiate into the trophectoderm (TE), *Oct4* expression becomes downregulated and restricted to cells of the ICM in the blastocyst [5, 7, 8]. When cells of the primitive endoderm differentiate and migrate away from the ectoderm, their Oct4 protein levels transiently increase [4]. Oct4 expression then becomes downregulated in the primitive endoderm and maintained in the epiblast, concurrently with embryo implantation and gastrulation. *Oct4* expression finally becomes restricted to PGCs [9], which are first specified in the extraembryonic mesoderm at the base of the allantoic bud during gastrulation [9]. PGCs give rise to gametes, which can be fertilized to develop into a new fully functional organism of the next generation and complete one cycle of life (Figure 1).

Oct4, encoded by the gene Pou5f1, is a homeodomain transcription factor of the POU (Pit-Oct-Unc) family. The POU family of transcription factors can activate the expression of their target genes through binding to an octameric sequence motif of an ATGCAAAT consensus sequence. Oct4 protein consists of 3 domains: N-terminal domain, POU domain, and a C-terminal domain. The POU domain consists of two structurally independent subdomains: a 75 amino acid amino-terminal POU-specific (POU_S_) region and a 60 amino-acid carboxyl-terminal homeodomain (POU_HD_). Both domains make specific contact with DNA through a helix-turn-helix structure and are connected by a linker of 17 amino acids. Regions outside of the POU domain are not critical for DNA binding and exhibit little sequence conservation. The N-terminal domain (N-domain) is rich in Proline and acidic residues, while the C-terminal domain (C-domain) is rich in Proline, Serine, and Threonine residues. Both the N-domain and the C-domain play an important role in transactivation, but the activity of the C-domain is cell type specific and is regulated through phosphorylation, whereas that of the N-domain is not. The Oct4 POU-domain functions differently by serving as an interaction site for binding by cell type–specific regulatory factors [12, 13]. *Oct4* has been deemed to be a critical regulator of cellular pluripotency, as shown by a zygotic *Oct4*-knockout study [14]. Loss of pluripotency in embryos, observed at the onset of somitogenesis, is coincident with reduction of *Oct4* and *Nanog* expression, and can be rescued by ectopic Oct4 expression [15]. *Oct4* can activate its own expression with its transcription factor partner *Sox2* through a positive autoregulatory loop in ESCs [16]. Studies on Oct4 interaction protein network have revealed that the Oct4 interactome includes many transcription factors and chromatin-modifying complexes with documented roles in self-renewal and pluripotency, and that acute depletion of Oct4 reduces the binding of Tcfcp2l1, Dax1, and Esrrb to target genes [17–19]. Depletion of Oct4 by siRNA leads to reduced binding of two key components of the bone morphogenetic protein (BMP) and leukemia inhibitory factor (LIF) signaling pathways, Smad1 and STAT3, to their respective targets. This result indicates that Oct4 plays a pivotal role in stabilizing the nucleoprotein complex and establishes a hierarchy of regulatory interactions between Oct4, STAT3, and Smad1 [20]. The core components of the pluripotency circuitry are formed by Oct4, Nanog, and Sox2, and *Nanog* expression is directly regulated by Oct4 and Sox2 [21], Sox2 is actually dispensable for the activation of Oct-Sox enhancers, and the forced expression of Oct4 could rescue Sox2-null ESCs [22]. Hence, *Oct4* is considered to be the genetic "master switch" in the establishment of totipotency-pluripotency during the life cycle of mammals [23], and it is presumed to be the most upstream gene in the molecular circuitry of pluripotency [24].

### Maternal Oct4 expression is not critical for developmental competence of the oocyte

All biological processes occurring during the first cell cycle of the embryo rely on maternal factors, which have accumulated during the long growth phase of the germinal vesicle (GV) oocyte [25], because there is nearly no mRNA synthesis between the end of the mouse oocyte growth phase and the first zygotic cleavage [26]. But during the second cell cycle, there is a burst of transcription—what is known as zygotic genome activation (ZGA) [27]. Mature metaphase II (MII) oocytes have the capacity to reprogram somatic cells into cells of a totipotent state via nuclear transfer (NT) [28, 29]. Therefore, activation of zygotic transcripts mediated by maternal regulatory factors provides the first step in the establishment of totipotency/pluripotency. *Oct4* is one of the 27 proven maternal-effect genes and is regarded to be functionally important for zygotic genome activation [30]. Maternal Oct4 is therefore widely accepted to play a role in igniting the establishment of totipotency and induction of pluripotency.

According to the chromatin organization, growing mouse oocytes could be classified into 2 types: SN (surrounded nucleolus) oocytes, with a ring of chromatin surrounding the nucleolus, and NSN (not surrounded nucleolus) oocytes, with chromatin dispersed throughout the nucleus, i.e., chromatin not surrounding the nucleolus. When they mature into metaphase II (MII) oocytes and are fertilized *in vitro*, only MII^SN^ oocytes (MII oocytes derived from SN oocytes) are developmentally competent to develop beyond the 2-cell stage, while MII^NSN^ oocytes (MII oocytes derived from NSN oocytes) become arrested at the 2-cell stage [31]. By comparing the transcriptional profiles of these two types of mouse MII oocytes, Zuccotti et al. found that Oct4 was absent in MII^NSN^ oocytes, accounting for the downregulation of *Stella*, a maternal-effect factor required for the oocyte-to-embryo transition, and the upregulation of 18 Oct4-regulated genes implicated in the activation of adverse biochemical pathways such as oxidative phosphorylation, mitochondrial dysfunction, and apoptosis. Those authors concluded that the downregulation of Oct4 leads to the developmental arrest of MII^NSN^ oocytes and that maternal Oct4 emerges as a key regulator of the molecular events that govern the establishment of the developmental competence of mouse oocytes [32]. Moreover, another study claimed that Oct4 is a critical regulator of the maternal-embryonic transition at the 2-cell stage, as embryos invasively injected with Oct4-antisense morpholino oligonucleotides were found to arrest in various developmental stages prior to the blastocyst stage [33]. A later report from the same group using the same approach also showed developmental arrest between the 2- to 8-cell stages in morpholino-mediated Oct4, Nanog, or Sall4 knockdown, but developmental arrest remained obvious after co-injection of Oct4, Nanog, or Sall4 mRNA [34]. Although such a phenotype contradicts to genetic knockout studies [14, 35, 36], which indicated that homozygous null embryos could develop at least up to the blastocyst stage, the authors ignored the presence of maternal Oct4 protein and assumed that the apparent discrepancy was due to the presence of functional maternal transcript [34]. However, recently, numerous studies found that after genetic removal of maternal Oct4 in oocytes by crossing *Oct4* ^*flox/flox*^ */ZP3* ^*Cre/+*^female mice with wild-type male mice, these *Oct4* ^*flox/flox*^ */ZP3* ^*Cre/+*^female mice were found to be fully fertile [37–39]. All offspring had deletion of the maternal *Oct4* allele as confirmed by polymerase chain reaction (PCR) genotyping, clearly demonstrating that maternal *Oct4* is not critical for the establishment of totipotency-pluripotency [37]. The data in the study also showed the efficient deletion of the maternal Oct4 allele by genotyping of individual oocytes, the depletion of Oct4 mRNA by real-time reverse transcriptase PCR (RT-PCR), and the depletion of Oct4 protein by Western blot [37].

### Oct4-null oocytes can reprogram somatic cells to a pluripotent state

Enucleated oocytes can also reprogram the nuclei of terminally differentiated somatic cells to a totipotent embryonic state after nuclear transplantation [28, 29]. The nuclei can be reprogrammed by the recipient oocytes to express the pluripotent gene *Oct4* at a very high efficiency (88.7%) in just 2–3 days [40] and to give rise to pluripotent cells at an efficiency of up to 20% [41]. This compares favorably with the exciting 4-factor (Oct4, Sox2, Klf4, and c-Myc) technique of generating induced pluripotent stem cells (iPSCs) by Yamanaka and colleagues, which showed activation of internal *Oct4* expression after more than 2 weeks, with an efficiency of 0.01–0.1% [42]. A recent study revealed that Oct4 alone could reprogram neural stem cells into pluripotent iPSCs [43]. Could maternal *Oct4* in oocytes be essential in the reprogramming of somatic cells to pluripotent status? The answer is no. By conducting NT experiments using maternal *Oct4*-null oocytes from *Oct4* ^*flox/flox*^ */ZP3* ^*Cre/+*^female mice, it was found that all cloned embryos expressed pluripotency genes (*Oct4* and *Nanog*) in the ICM and the TE marker gene *Cdx2* in the TE by both immunocytochemistry and real-time RT-PCR [37]. Furthermore, ESC lines derived from cloned embryos using *Oct4*-knockout oocytes demonstrated full pluripotency by generating completely ESC-derived mice through the tetraploid complementation test, the most stringent test for pluripotency [37]. Clearly, the reprogramming engine in oocytes could work effectively to reprogram somatic cells into cells of a pluripotent state without the presence of maternal Oct4.

### Reciprocal interaction between Oct4 and Cdx2 is not the initial cause sparking ICM/TE lineage separation

Previous studies have suggested that lineage commitment is controlled by the expression level of *Oct4*[14, 44]. Repression of *Oct4* expression induced the differentiation of ESCs into TE cells, and a less than two-fold increase in *Oct4* expression caused the differentiation of ESCs into cells of primitive endoderm and mesoderm [44]. In the absence of Oct4, embryos could not form an ICM—i.e., the inner cells of morula-stage embryos rather were driven into trophoblast differentiation [14]. Therefore, these results indicated that *Oct4* plays a critical role in sustaining stem cell self-renewal and that up- or downregulation of *Oct4* expression induces divergent developmental programs, suggesting that *Oct4* is the master regulator of pluripotency and that it may also control lineage commitment during early embryonic development [14]. This hypothesis was supported by reports showing that Oct4 and Cdx2 could form a complex that reciprocally repressed their target genes and such an interaction determined the ICM/TE lineage separation in early embryos [45], while the interaction between Oct4 and the histone H3–specific methyltransferase ESET restricted the extraembryonic trophoblast lineage potential of pluripotent cells [46]. However, the expression of *Cdx2* in ESCs was found to be rapidly initiated by Ras activation (i.e., within 24 hours) without previous or simultaneous downregulation of *Oct4* expression [47], while ESCs with reduced Oct4 expression showed robust pluripotency and expressed naïve pluripotency genes, but were deficient in differentiation in the absence of pluripotency culture requisites [48, 49]. On the other hand, *Cdx2* deficiency did not interrupt TE-ICM lineage separation [50–52], and zygotic *Oct4* expression was shown not to be required for the initial repression of the TE genes *Cdx2* and *Gata3* in the ICM, indicating that other mechanisms are responsible for restricting the expression of these genes to the TE [53]. Finally, with a conditional knockout system, it was demonstrated that, similar to the reported zygotic Oct4 knockout observation [14, 53], the genetic removal of both maternal and zygotic Oct4 did not prevent ICM-TE lineage separation [37–39], arguing against the notion that maternal Oct4 could partially compensate for the loss of zygotic Oct4 during cell fate specification in the blastocyst [33]. Therefore, the first lineage separation of the ICM-TE is not determined by the reciprocal interaction between Oct4 and Cdx2. More importantly, these studies indicated that maternal Oct4 is not at the root of pluripotency as a determinant of pluripotent cell lineage initiation, contrary to previous assumptions and views [23, 24].

### How Oct4 is activated in the embryo remains an open question

Oct4 is at the top of the pluripotency regulatory hierarchy in pluripotent cells [20, 21]. It forms a positive feedback loop [16] and is essential to maintaining pluripotency [14], but is not required for initiating totipotency/pluripotency in embryos [37–39]. Therefore, understanding how early embryos activate *Oct4* expression is key in clarifying the oocyte reprogramming mechanism.

**Figure 2:**
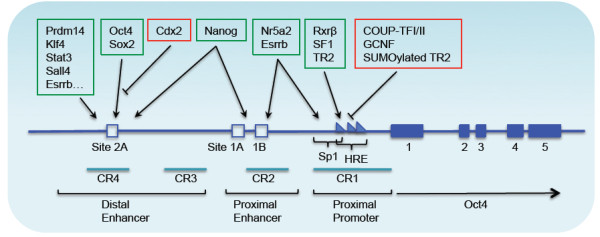
**Genomic structure and transcriptional regulation of the mouse** ***Oct4*** **gene.** The diagram represents ~24 kb of the genomic region surrounding the *Oct4* gene [62]. The gene has five exons, depicted as blue boxes. The identified upstream regulatory regions include the promoter, proximal enhancer, and distal enhancer. The sizes of the regulatory elements are stretched to enhance clarity. The transcription factors bind to these regions, and are shown above within colored boxes; they either activate (green box) or repress (red box) transcription. HRE = hormone responsive element; Sp1 = GC-rich site recognized by the Sp1/Sp3 family of transcription factors. CR1, CR2, CR3, and CR4 are conserved regions (CRs) at the 5’ upstream region of the *Oct4* gene.

The upstream region of the transcriptional initiation site of the *Oct4* gene contains three regulatory elements for gene transcription: the distal enhancer (DE), proximal enhancer (PE), and TATA-less proximal promoter (PP) [9] as illustrated in Figure 2. The two enhancers exhibit a differential activation pattern according to the developmental stage of the mouse embryo. The DE drives *Oct4* expression in the ICM, ESCs, and PGCs, while the PE activates *Oct4* expression in epiblast cells. Each enhancer contains multiple potential binding sites for transcription factors that can either activate or repress *Oct4* expression. In addition, the methylation of these regions represses *Oct4* expression in differentiated cells. Several positive and negative regulators bind to the *Oct4* gene to regulate its expression. Of these, members of the orphan nuclear receptor superfamily, which can bind to Sp1 sites [54] and hormone response elements (HREs) in the PE and PP, are known to influence *Oct4* expression. Positive regulators of *Oct4* expression include *Nr5a2*[55], *SF1* (Steroidogenic Factor-1), and *RXR-β* (Retinoid X Receptor-β, also known as Nr2b2) [56, 57]. Negative regulators include *GCNF* (Germ Cell Nuclear Factor) (also known as *Nr6a1*) [58], and *COUF-TFI/II* (Chicken Ovalbumin Upstream promoter-Transcription Factors- I/II), encoded by *Nr2f1* and *Nr2f2*, respectively [59, 60]. The transcription factor TR2 can bind to the HRE of the *Oct4* gene to either activate or repress *Oct4* expression in P19 embryonal carcinoma (EC) stem cells and regulate the proliferation of the culture, based on whether there is SUMOylation on the Lys-238 of TR2 [61].

Recent studies have found that certain maternal factors are involved in the regulation of *Oct4* expression, providing clues on the mechanism underlying the initiation of totipotency/pluripotency. Cancer-associated factor Tpt1 has been reported to activate the transcription of *Oct4* and *Nanog* in transplanted somatic nuclei in the Xenopus oocyte [63], but another study failed to replicate this finding upon knockdown of Tpt1 by Small interfering RNA (siRNA) in the mouse embryos [37]. Components of the ATP-dependent BAF chromatin-remodeling complex have been shown to significantly increase reprogramming efficiency when used together with the Yamanaka’s 4 factors [64]. Promyelocytic leukemia (Pml) protein was found to be required for *Oct4* gene expression and the maintenance of its open chromatin conformation in stem cells. In proliferating stem cells, Pml-nuclear body, along with the transcription factors TR2, SF1 and Sp1, and the Brg1-dependent chromatin remodeling complex (BRGC), associates with the Oct4 promoter to maintain a nucleosome-free region for gene activity [65]. Studies in search for master genes in the oocyte have revealed a novel oocyte-specific eukaryotic translation initiation factor 4E (*Eif4eloo*) [66] and a large number of oocyte-specific genes with yet unknown functions, such as those belonging to the homeodomain transcription factor Obox family [67]. The maternal transcription factor Sall4 binds to the *Oct4* DE, and it is regarded to be a transcriptional activator of *Oct4* expression based on evidence that reduction in *Sall4* mRNA level in blastocysts at merely 50% knockdown efficiency by *Sall4* siRNA injection into zygotes led to a 70% reduction in *Oct4* expression [68]. Single-cell expression analyses during *in vitro* cellular reprogramming have confirmed that *Sall4* is indeed an upstream activator of *Oct4* expression [69]. However, the efficient knockdown of *Sall4* by injecting *Sall4* siRNA into maternal *Oct4*-deficient zygotes—to avoid any possible effect of maternal Oct4 as a positive autoregulator—did not lead to any changes in *Oct4* expression at the blastocyst stage, arguing against such a role for *Sall4* as an upstream activator of *Oct4* expression *in vivo*[37]. Nr5a2 was found to maintain Oct4 expression at the epiblast stage of embryonic development, by binding to the PE and PP regions of *Oct4*, but to play no evident role in ESC self-renewal [55]. However, Nr5a2 can induce epiblast stem cells into ground state pluripotency, a basal proliferative state that is free of epigenetic restriction [70], and replace Oct4 in the reprogramming of somatic cells into pluripotent cells [71]. Activation of *Zscan4* expression occurs during ZGA, with the gene being expressed in ESCs, whereas reduction in *Zscan4* transcript levels by siRNAs delays the progression from the 2-cell to the 4-cell stage, leading to blastocysts that fail to implant or proliferate in blastocyst outgrowth culture [72]. Zscan4 is essential for induction of iPSCs and its ectopic expression can activate early embryonic genes and improve the efficiency of iPSC generation [73]. However, knockdown of *Zscan4* in preimplantation embryos by siRNA against all 6 isoforms of *Zscan4* (*a-f*) had no impact on *Oct4* expression [37]. A component of an active DNA demethylase, activation-induced cytidine deaminase (*AID*), was also shown to be required for reprogramming [74]. A genome-scale RNA interference (RNAi) screen in ESCs identified components of the Paf1 complex with strong effects on *Oct4* expression, and showed that Paf1C overexpression blocks the differentiation of ESCs and that Paf1C knockdown causes expression changes in ESCs that are similar to those observed with Oct4 or Nanog depletion [75].

### Oct4B expression in oocytes and embryos

**Figure 3:**
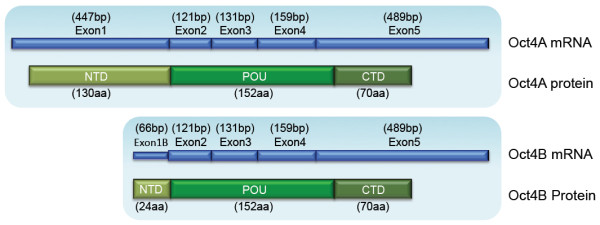
**Schematic representation of the protein domains of the mouse Oct4 isoforms and the corresponding exons.** Exon1B of Oct4B is in the intron 1–2 region of the *Oct4* gene. Modified from Guo et al. 2012 [81].

After the finding that truncated isoforms of OCT4 are transcribed from the POU5F1 gene in human [76] and mouse [77], the originally described OCT4 is designated as OCT4A and the newly found truncated isoforms are variants of an OCT4 version named OCT4B [78]. OCT4B mRNAs encode proteins that have identical POU DNA-binding domains and C-domains but differ in their N-domains (Figure 3). Continued expression of Oct4B after the original Oct4 promoter was removed indicates that Oct4B transcription is regulated by an alternative promoter in the first intron [37], as presumed by an earlier study [79]. Other isoforms of Oct4B could be produced by alternative splicing or alternative translation initiation [80, 81]. ESC-based complementation assays using ZHBTc4 ESCs, which has endogenous Oct4 inactivated by gene targeting and harbors a tetracycline-repressible Oct4 transgene to support ESC self-renewal [44], showed that OCT4B cannot rescue the self-renewal ability of ZHBTc4 ESCs in the presence of doxycycline, unlike OCT4A. Electrophonetic mobility shift assay showed that OCT4B does not bind to a probe carrying the OCT4 consensus binding sequence due to the repressive effect of the OCT4B N-domain. Furthermore, overexpression of OCT4B does not activate transcription from OCT4-dependent promoters [78]. However, Oct4B is involved in stress response [82] and acts as an antiapoptotic factor in cancer cells [83].

On the other hand, Oct4 expression in the adult has been reported in hematopoietic and mesenchymal stem cells [84–95], as well as progenitor cells from various somatic tissues including pancreatic islets [96], kidney [97, 98], peripheral blood [89, 99, 100], endometrium of the uterus [101, 102], thyroid [103], lung [104], brain [105, 106], liver [107], and skin [108–111]. The wide expression of Oct4 in normal tissues suggests that *Oct4* may not only be crucial for the maintenance of pluripotency in embryonic cells, but also play an important role in the self-renewal of somatic stem cells and in maintenance of tissue homeostasis. As these studies did not distinguish the Oct4 isoforms, it is likely they actually detected Oct4B. A later study that conditionally deleted Oct4 from somatic cells *in vivo* found that Oct4 is dispensable for both the self-renewal and maintenance of somatic stem cells in the adult mammal [112]. Oct4 gene ablation in the intestinal epithelium, bone marrow (hematopoietic and mesenchymal lineages), hair follicle, brain, and liver revealed no abnormalities in homeostasis or regenerative capacity [112]. Like many other publications claiming Oct4 expression in somatic stem cells, this study also noted low level of expression but that was regarded as false detection of Oct4 due to the noise of the detection methods, expression of pseudogenes, and expression of other POU-domain family members [112]. These conclusions are now in question. In our lab, the same Oct4floxed mice were used, in which two *LoxP* motifs had been inserted that span the proximal promoter and the Oct4A-unique first exon [2]. As the other 4 exons shared by *Oct4B* were not mutated, it is still possible that *Oct4*B can still be transcribed using an alternative promoter. After efficiently removing the floxed sequence of *Oct4* in oocytes by crossing the *Oct4* ^*flox/flox*^ mice with ZP3Cre transgenic mice, indeed, the expression of only Oct4B (not Oct4A) was detected at low levels by RT-PCR in the mutated oocytes and preimplantation embryos, which was confirmed by sequencing. Another recent report used a different Oct4floxed mouse line to delete the entire Oct4 POU domain and C-domain, yet the authors of that study also observed full-term development of maternal Oct4–null embryos and TE/ICM lineage separation, as well as Nanog activation in maternal and zygotic Oct4–null embryos [39]. Taken together, these studies confirmed that (1) maternal Oct4 is indeed not essential for the establishment of totipotency. (2) The low levels of Oct4B expression could not rescue Oct4A-null embryos to maintain pluripotency *in vivo*[37, 38]. The precise function of Oct4B in embryos and somatic stem cells remains to be clarified.

## Conclusion

**Figure 4:**
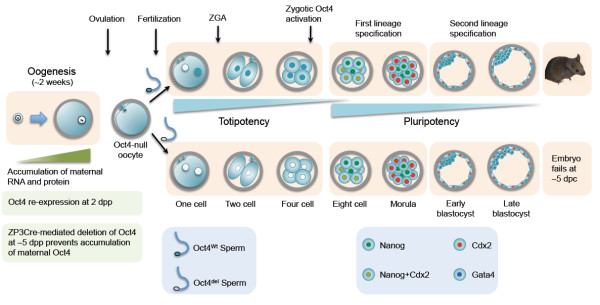
**Development of maternal Oct4–depleted embryos.** Totipotency in maternal Oct4–depleted embryos can be established in the absence of Oct4, and these embryos can maintain pluripotency and complete full-term development, supported by the zygotic activation of the paternal allele Oct4 gene at the late 4-cell stage. The lower panel shows that in the absence of both maternal and zygotic Oct4 expression, the Nanog-positive ICM and Cdx2-positive TE lineages are still established. However, this ICM cannot maintain pluripotency and complete the second lineage separation, and it fails to further develop at around the time of implantation. dpc: days post coitum; dpp: days post partum; ZGA: zygotic genome activation.

As summarized in Figure 4, new pieces of evidence clearly indicate that Oct4 is not the master regulator responsible for initiating totipotency-pluripotency in oocytes, and that maternal and zygotic Oct4–null blastocysts maintain the ability to activate Nanog and Oct4-GFP expression, indicating that unknown pathways other than the Oct4-centered pluripotency-regulating network are active in embryos and function upstream of Oct4 in driving pluripotency. However, to date no factors have proven to be essential for Oct4 activation in the preimplantation embryos. Further studies are required to elucidate how oocytes activate the pluripotent genes *Oct4* and *Nanog* on top of the Oct4/Sox2 autoregulatory loop in an effort to understand the establishment of totipotency in zygotes and in transplanted somatic cells.

## Abbreviations

ICM: Inner cell mass
ESCs: Embryonic stem cells
TE: Trophectoderm
PGCs: Primordial germ cells
Pit-Oct-Unc: POU
POUS: POU-specific
POUHD: Homeodomain
N-domain: N-terminal domain
C-domain: C-terminal domain
BMP: Bone morphogenetic protein
LIF: Leukemia inhibitory factor
GV: Germinal vesicle
ZGA: Zygotic genome activation
MII: Metaphase II
SN: Surrounded nucleolus
NSN: Not surrounded nucleolus
NT: Nuclear transfer
PCR: Polymerase chain reaction
RT-PCR: Reverse transcriptase PCR
iPSCs: Induced pluripotent stem cells
DE: Distal enhancer
PE: Proximal enhancer
PP: Proximal promoter
HREs: Hormone response elements
SF1: Steroidogenic Factor-1
RXR-Beta: Retinoid X Receptor-Beta
GCNF: Germ Cell Nuclear Factor
COUF-TFI/II: Chicken Ovalbumin Upstream promoter-Transcription Factors- I/II
BRGC: Brg1-dependent chromatin remodeling complex
Eif4eloo: Oocyte-specific eukaryotic translation initiation factor 4E
AID: Activation-induced cytidine deaminase
siRNA: Small interfering RNA
RNAi: RNA interference
CRs: Conserved regions
dpc: Days post coitum
dpp: days post partum.
